# Longitudinal ozone exposure and SARS-CoV-2 infection in late pregnancy: a retrospective cohort study

**DOI:** 10.3389/fcimb.2024.1476603

**Published:** 2024-12-20

**Authors:** Lin Zhang, Jiaqi Tian, Shuyin Duan

**Affiliations:** ^1^ Clinical Medical Research Center for Women and Children Diseases, Key Laboratory of Birth Regulation and Control Technology of National Health Commission of China, Shandong Provincial Maternal and Child Health Care Hospital Affiliated to Qingdao University, Jinan, China; ^2^ Key Laboratory of Birth Defect Prevention and Genetic Medicine of Shandong Health Commission, Jinan, China; ^3^ School of Public Health, Shandong First Medical University & Shandong Academy of Medical Sciences, Jinan, China

**Keywords:** gestational ozone exposure, SARS-CoV-2 infection, pregnancy outcomes, environmental epidemiology, viral susceptibility

## Abstract

**Background:**

Atmospheric ozone is a common air pollutant with known impacts on maternal and fetal health. However, the relationship between gestational ozone exposure and susceptibility to respirovirus infection remains unclear. This study aims to assess the association between longitudinal ozone exposure during pregnancy and COVID-19 risk in late gestation.

**Methods:**

This retrospective cohort study included 600 pregnant women (300 infected with SARS-CoV-2 and 300 uninfected) who delivered at one hospital in Jinan, China from November 2022 to January 2023. Individual ozone exposure levels throughout gestation were estimated based on local ambient monitoring data. Logistic regression models were constructed to examine the association between gestational ozone exposure and COVID-19 status in late pregnancy, adjusting for demographic and clinical variables.

**Results:**

In Jinan, ozone levels increased by 1.85 ppb annually and peaked between April and October, corresponding to winds from the south and southwest. Higher ozone exposure over pregnancy was associated with lower odds of SARS-CoV-2 infection in late gestation (OR per IQR increase = 0.60, 95% CI: 0.40-0.90). Compared to the lowest quartile (reference), the highest ozone quartile corresponded to a 99% decreased infection risk (OR = 0.01, 95% CI: 0.00-0.03). Increased maternal age and pre-pregnancy BMI were associated with higher COVID-19 risk.

**Conclusions:**

Longitudinal ozone exposure during gestation may be protective against SARS-CoV-2 infection in late pregnancy. Further studies are needed to confirm this finding and elucidate underlying mechanisms. These results highlight the potential importance of environmental exposures in COVID-19 susceptibility.

## 1 Introduction

The global pandemic of coronavirus disease 2019 (COVID-19), caused by the severe acute respiratory syndrome coronavirus 2 (SARS−CoV−2) (one newly defined respirovirus), has impacted populations globally since its emergence in late 2019. With the advent of new viral variants, repeat infections are becoming increasingly prevalent, and there is growing evidence to suggest that these may heighten the risk of developing long COVID (Adrielle dos Santos et al., 2021; Cohen and Pulliam, 2023).

Pregnant women are typically more susceptible to infections due to physiological changes during pregnancy, and they represent a particularly vulnerable group during such infectious disease outbreaks. In fact, COVID-19 has been shown to result in more severe morbidity in pregnant women as compared to non-pregnant populations (Schwartz, 2020; Wastnedge et al., 2021). Increasing evidence has demonstrated adverse outcomes associated with SARS-CoV-2 infection during pregnancy, which includes preterm birth, stillbirth, and maternal mortality (Chmielewska et al., 2021; Khalil et al., 2020). In addition to understanding the implications of respirovirus with vertical transmission, such as Zika virus, which has led to significant fetal health complications, it is important to consider SARS-CoV-2, a novel respiratory virus that also raises concerns regarding potential vertical transmission (Cervantes et al., 2023). The evidence for SARS-CoV-2 vertical transmission is still evolving, but studies have indicated possible transmission from mother to fetus (Ketabi et al., 2023; Wei et al., 2024). Consequently, the identification of risk factors for repeat infections in pregnant women has emerged with the highest public health priority for mitigating both immediate impacts and long-term sequelae such as long COVID.

One potential risk factor for COVID-19 is exposure to air pollution (Hernandez Carballo et al., 2022; Jerrett et al., 2023; Wen et al., 2022). As a dominant component of air pollutants, the ground-level ozone is usually formed by chemical reactions between nitrogen oxides, volatile organic compounds, methane, and carbon monoxide in the presence of sunlight (Hien et al., 2022; Lyu et al., 2022; Zheng et al., 2022). However, ozone exposure during pregnancy has been shown to cause oxidative stress, inflammation, and endothelial dysfunction (Mozzoni et al., 2022). At the molecular level, ozone can impair placental growth factor signaling and increase anti-angiogenic factors, which is causally associated with dysregulations of placental vascular development (Hunter et al., 2023). Numerous studies have linked gestational ozone exposure with adverse birth outcomes, including low birth weight, small for gestational age, and preterm birth (Chen et al., 2023; Sun et al., 2022). However, the mechanisms linking ozone exposure and adverse pregnancy outcomes are not fully understood.

Although previous studies have examined the impacts of ozone exposure in pregnancy, few examined the relationship between longitudinal ozone exposure during pregnancy and SARS-CoV-2 infection in late pregnancy, and this represents a significant gap in the literature that requires immediate attention. It is plausible that ozone-induced placental dysfunction alters the susceptibility to viral infections, but more research is critically needed to investigate this hypothesis further. Therefore, in this retrospective cohort study, we aimed to assess the association between longitudinal ozone exposure over gestation and the risk of testing positive for SARS-CoV-2 in late pregnancy, which will help elucidate whether air pollution is a novel risk factor for COVID-19 in pregnant women and provide insights into the mechanisms linking ozone and SARS-CoV-2 infection.

## 2 Methods

### 2.1 Population

This study was conducted in Jinan, east China between November 3, 2022 and January 6, 2023, encompassing one month before and after the Chinese government easing of COVID-19 control measures. The study population consisted of 600 pregnant women with singleton births who were recruited during this period, of whom 300 were infected with SARS-CoV-2 and 300 were not. Detailed maternal information was extracted from electronic medical records and questionnaires, including age, pre-pregnancy body mass index (BMI), education background, vaccination status, gestational diabetes mellitus (GDM), folic acid supplementation, medication use during pregnancy, age at first pregnancy, gravidity, prior preterm births or abortions, age at menarche, menstrual cycle characteristics (days of menstruation, cycle length, regularity), gestational age at delivery, conception method, SARS-CoV-2 infection status, and infant sex. Exclusion criteria were set as follows: residence outside the study area, neonate congenital disabilities, and maternal age at delivery outside 18-50 years. All participants denied any history of alcohol, tobacco, or drug use. The study protocol was approved by the Ethics Committee of the Maternal and Child Health Care Hospital of Shandong Province Affiliated to Qingdao University in Jinan, China (NSFC.2022-023).

### 2.2 Exposure

In accordance with our previous study (Zheng et al., 2022), we monitored ground-level ozone in the designated study area on an hourly basis using publicly accessible data at the Air Quality and Pollution Measurement website (https://aqicn.org/), which is part of the World Air Quality Index project that commenced in 2007. We calculated the gestational atmospheric ozone exposure levels by averaging daily ozone concentrations from the onset of pregnancy until delivery. Simultaneously, we gathered data on local temperature, wind direction and wind speed from the NOAA Integrated Surface Database (ISD) via the worldmet package in R software. To examine the long-term trend of atmospheric ozone from 2015 to 2023, we conducted Theil-Sen trend statistical analysis, where the openair package was employed to analyze and illustrate variations in atmospheric ozone on an hourly and daily basis.

### 2.3 Outcomes and covariates

The primary outcome of this study was SARS-CoV-2 infection rate, which was detected through reverse transcription polymerase chain reaction (RT-PCR) testing of nasal and pharyngeal swabs according to the instructions provided by the commercial PCR kit. The assay targeted the ORF1ab, N, and E genes of the SARS-CoV-2 virus. Specific primer sequences used for amplification were as follows. RdRP, forward primer: GTGARATGGTCATGTGTGGCGG, reverse primer: CARATGTTAAASACACTATTAGCATA; N, forward primer: CACATTGGCACCCGCAATC, reverse primer: GAGGAACGAGAAGAGGCTTG; E, forward primer: ACAGGTACGTTAATAGTTAATAGCGT, reverse primer: ATATTGCAGCAGTACGCACACA.

For study subjects, we randomly recruited them at 29-40 gestational weeks. Covariates were chosen based on previous studies (Wang et al., 2022) and clinical experience, including gestational age, pre-pregnancy BMI, education background, vaccination status, GDM, folic acid supplementation, medication use during pregnancy, age at first pregnancy, gravidity, prior preterm births or abortions, age at menarche, menstrual cycle days and length, gestational age at delivery, conception method, and infant sex. Of them, gestational age, pre-pregnancy BMI, age at first pregnancy, age at menarche, menstrual cycle length, and gestational age at delivery were continuous variables, while the education background, vaccination status, GDM, folic acid supplementation, medication use during pregnancy, menstrual cycle regularity, conception method, and infant sex were categorical variables. Noteworthy, we collected hourly atmospheric ozone measurements, calculated daily averages, then gestational averages, and included them as continuous variables in our analyses.

### 2.4 Statistical analysis

Continuous demographic variables and atmospheric ozone exposure data were presented as mean and standard deviation. Categorical variables were showed as frequency with corresponding percentage. All study subjects were divided into two groups based on the infection status, with 300 subjects each group. Differences between groups were described and compared using t-test, chi-square test, or Fisher’s exact test, as appropriate.

To determine the effect of gestational ozone exposure on SARS-CoV-2 infection during late pregnancy, we employed both univariate and multivariate logistic regression analyses. Initially, we explored the univariate relationship between each variable and SARS-CoV-2 infection utilizing logistic regression analysis. Subsequently, we adjusted for potential confounding effects introduced by demographic variables using a multivariate logistic regression model. Simultaneously, we restructured the ozone exposure data into increments of interquartile ranges and incorporated it as a categorical increment in an alternate multivariate model. Prior to multivariate modeling, we scrutinized the collinearity between variables using correlation analysis (**Supplementary Figure S1**). All analyses were executed in R 4.3.1. A p-value of less than 0.05 was deemed statistically significant unless stated otherwise.

## 3 Results

### 3.1 Variation of atmospheric ozone of the study area

**Figure 1:**
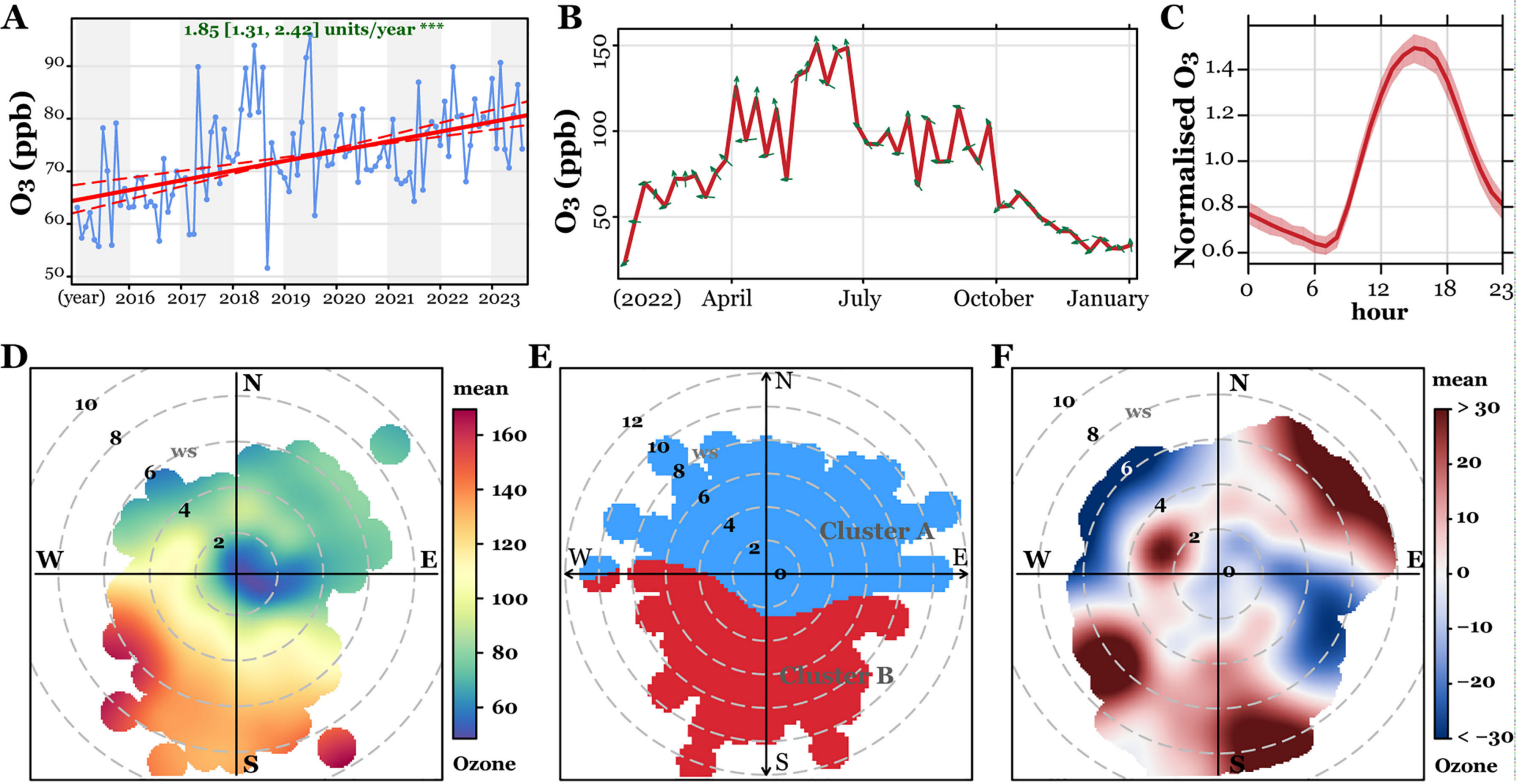
Characterization of atmospheric ozone in Jinan, China between 2015 and 2023. **(A)** Theil-Sen trend analysis of ozone levels from 2015 to 2023. **(B)** Monthly ozone variations for 2022 and 2023, with wind directions indicated by green arrows. **(C)** Hourly ozone variations throughout the study period. **(D–F)** Polar plots illustrating ozone level variations **(D)**, clustering analysis **(E)**, and differences between 2021 and 2022 **(F)**.

To characterize the atmospheric ozone of the study area, we studied its longitudinal change from 2015 to 2023. As shown in **Figure 1**, the Theil-Sen regression analysis revealed that the atmospheric ozone continuously grew at 1.85 ppb/year, ranging from 32.67 to 95.54 ppb over the study period. Besides, the annual atmospheric ozone peaked between April and October, at which time the wind blew from the south or southwest. Similarly, the daily ozone concentration changed in line with temperature, which was detected with the highest concentration at 14:00 in the noon and the lowest at 7:00 in the morning. Based on ozone data collected from each pregnancy period, we showed its dynamic variation through polar plots, where we defined the highest ozone concentration at wind directions of south and west, with relatively high local temperature in summer. Consistently, the cluster analysis identified two clusters of the high and low ozone clusters, and compared to the year 2021, the atmospheric ozone increased in 2022 with wind blowing from the south, southwest, and northeast, suggesting that the ozone of Jinan is worsening both in summer and winter. These data suggest that the atmospheric ozone in Jinan is worsening and changes tightly and periodically with local temperature.

### 3.2 Baseline characteristics of study subjects

A total of 600 pregnant women with singleton birth were included in this study (**Table 1**), the age of whom varied between 19 and 47 years, with an average of 30.91 ± 4.26 years. Pre-pregnancy BMI values were also recorded, averaging at 26.81 ± 4.00 and spanning from 13.72 to 41.77. The women’s first pregnancies typically occurred around the age of 27.41 ± 3.63 years, with individual cases ranging from 16 to 42 years. Menarche was reported to have started at an average age of 13.79 ± 1.26 years, with a range of 11 to 32 years across the group. Menstrual cycles generally lasted for about 5.74 ± 0.91 days, with individual cycles ranging from 2 to 7.5 days in length. The gestational age at delivery averaged at 39.10 ± 2.07 weeks, with individual cases ranging from 32.00 to 41.43 weeks. Lastly, gestational ozone exposure levels were measured, averaging at 88.64 ± 3.34 ppb and ranging from 32.67 to 95.54 ppb across the group.

**Table 1:** Description and comparison of baseline characteristics between groups.

Variables	All (N=600)	Uninfected (N=300)	Infected (N=300)	Odds Ratio	p-value
Age at pregnancy (yrs)	30.91 ± 4.26	30.73 ± 4.20	31.08 ± 4.33		0.311
Height (cm)	163.03 ± 5.25	163.29 ± 5.13	162.77 ± 5.37		0.223
Weight (kg)	71.26 ± 11.36	70.42 ± 11.03	72.09 ± 11.63		0.072
Pre-pregnancy BMI (kg/m^2^)	26.81 ± 4.00	26.41 ± 3.88	27.20 ± 4.09		0.015
Education background (n)					0.818
Primary school	20 (3.33%)	12 (4.00%)	8 (2.67%)	Ref.	
High school	48 (8.00%)	24 (8.00%)	24 (8.00%)	1.48[0.51;4.48]	
University Graduate	450 (75.00%)	222 (74.00%)	228 (76.00%)	1.53[0.61;4.02]	
Postgraduate	82 (13.67%)	42 (14.00%)	40 (13.33%)	1.42[0.52;4.02]	
Vaccination (n)					0.035
No	126 (21.00%)	74 (24.67%)	52 (17.33%)	Ref.	
Yes	474 (79.00%)	226 (75.33%)	248 (82.67%)	1.56[1.05;2.33]	
GDM (n)					0.047
No	427 (71.17%)	225 (75.00%)	202 (67.33%)	Ref.	
Yes	173 (28.83%)	75 (25.00%)	98 (32.67%)	1.45[1.02;2.08]	
Folic acid supplementation (n)					0.860
No	34 (5.67%)	16 (5.33%)	18 (6.00%)	Ref.	
Yes	566 (94.33%)	284 (94.67%)	282 (94.00%)	0.88[0.44;1.78]	
Medication (n)					0.558
No	549 (91.50%)	277 (92.33%)	272 (90.67%)	Ref.	
Yes	51 (8.50%)	23 (7.67%)	28 (9.33%)	1.24[0.69;2.23]	
Age at first pregnancy (yrs)	27.41 ± 3.63	27.55 ± 3.86	27.27 ± 3.39		0.345
Gravidity (n)					0.125
0	250 (41.67%)	136 (45.33%)	114 (38.00%)	Ref.	
1	250 (41.67%)	121 (40.33%)	129 (43.00%)	1.27[0.89;1.81]	
≥2	100 (16.67%)	43 (14.33%)	57 (19.00%)	1.58[0.99;2.53]	
Preterm birth (n)					0.092
0	587 (97.83%)	297 (99.00%)	290 (96.67%)	Ref.	
≥1	13 (2.17%)	3 (1.00%)	10 (3.33%)	3.29[0.98;15.52]	
Abortion (n)					1.000
0	569 (94.83%)	285 (95.00%)	284 (94.67%)	Ref.	
≥1	31 (5.17%)	15 (5.00%)	16 (5.33%)	1.07[0.51;2.24]	
Age at menarche (yrs)	13.79 ± 1.26	13.91 ± 1.56	13.68 ± 0.84		0.027
Menstrual days (days)	5.74 ± 0.91	5.64 ± 0.99	5.84 ± 0.81		0.009
Menstrual Cycle (days)	31.56 ± 9.81	31.20 ± 7.42	31.92 ± 11.72		0.367
Menstrual Cycle regularity (n)					0.241
Regular	562 (93.67%)	277 (92.33%)	285 (95.00%)	Ref.	
Irregular	38 (6.33%)	23 (7.67%)	15 (5.00%)	0.64[0.32;1.24]	
Gestational age at delivery (wks)	39.10 ± 2.07	39.09 ± 2.61	39.11 ± 1.34		0.904
Conception method (n)					0.847
Artificial technology	28 (4.67%)	13 (4.33%)	15 (5.00%)	Ref.	
Natural pregnancy	572 (95.33%)	287 (95.67%)	285 (95.00%)	0.86[0.39;1.86]	
Sex of the newborn (n)					1.000
Male	305 (50.83%)	152 (50.67%)	153 (51.00%)	Ref.	
Female	295 (49.17%)	148 (49.33%)	147 (49.00%)	0.99[0.72;1.36]	
O3 (ppb)	88.64 ± 3.34	89.91 ± 4.03	87.37 ± 1.71		<0.001

In terms of categorical variables, a total of 580 subjects (80%) had a high school education or higher, 126 subjects (79%) received at least one vaccination, and 173 pregnant women were diagnosed with GDM, while 566 subjects received folic acid supplementation during their pregnancy. A total of 51 subjects took medication for conditions other than COVID-19 during their pregnancy, and while most subjects (572) conceived naturally, a minority (48) used artificial conception methods. Regarding the newborns, there were slightly more males (305) than females (295).

Based on whether they were infected with SARS-CoV-2, all study subjects were divided into two groups, with each group containing 300 subjects. Compared to the uninfected pregnant women, those infected with SARS-CoV-2 had a higher pre-pregnancy BMI, vaccination rate, prevalence of GDM, and menstrual days, but a lower age at menarche and gestational ozone exposure level. There were no statistically significant differences between groups in terms of the remaining demographic variables (p > 0.05).

### 3.3 Risk assessment of gestational ozone exposure on late pregnancy SARS-CoV-2 infection

**Figure 2:**
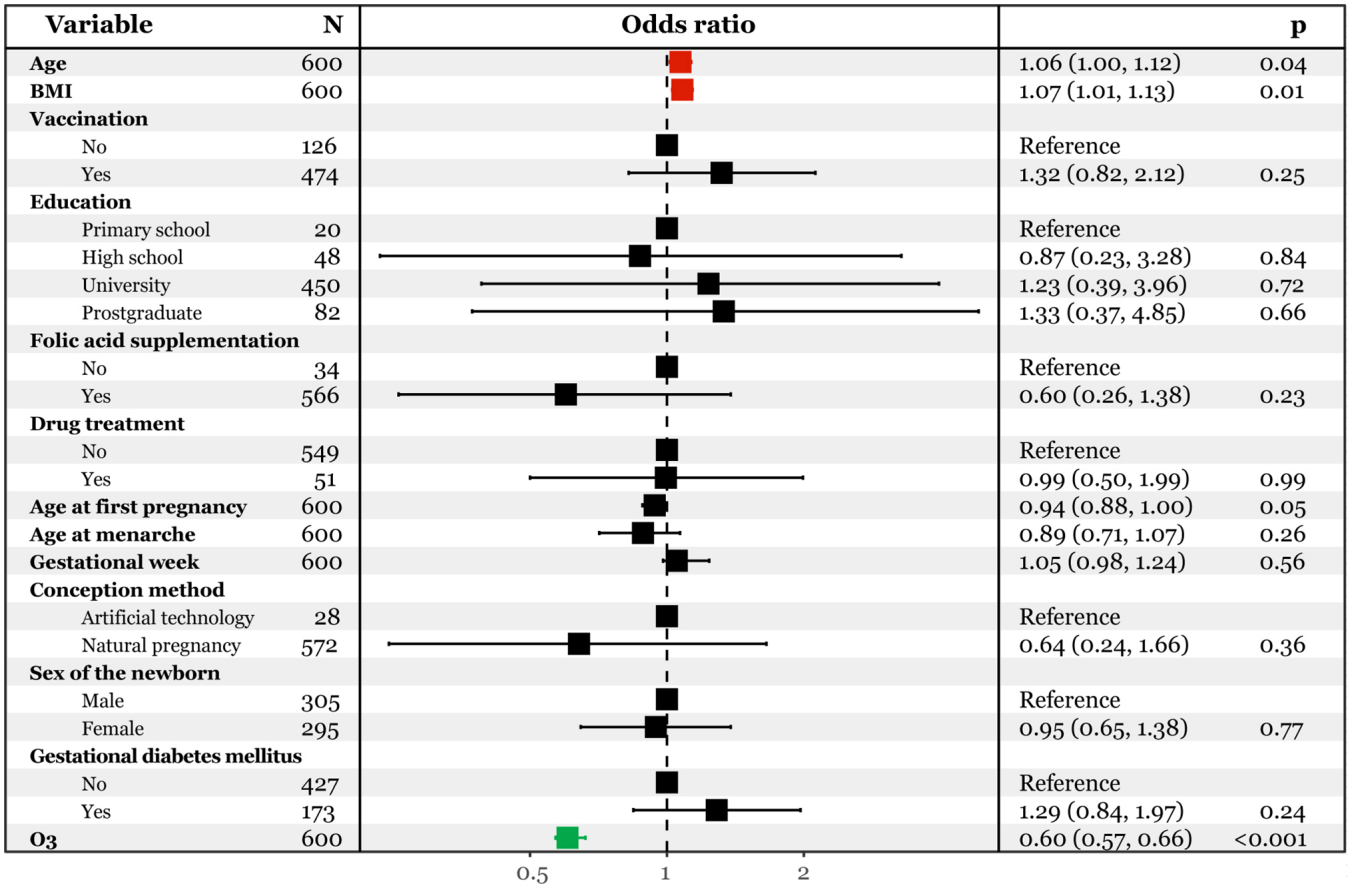
Multivariate logistic regression analysis predicting SARS-CoV-2 infection with continuous ozone.

To investigate the association of gestational ozone exposure and late pregnancy SARS-CoV-2 infection, we conducted multivariate logistic regression analyses. The model considered the infection status as the dependent variable (infected vs. uninfected), and demographic characteristics and gestational ozone exposure data as independent variables. As depicted in **Figure 2**, after adjusting for confounding effects of demographic variables, we found that each unit increase in gestational ozone exposure decreased 40% risk of late pregnancy SARS-CoV-2 infection [OR95%CI: 0.60(0.57, 0.66)]. Conversely, an increase in age at pregnancy and pre-pregnancy BMI was associated with an elevated risk of infection [OR95%CI: 1.06(1.00, 1.12) and 1.07(1.01, 1.13)].

**Figure 3:**
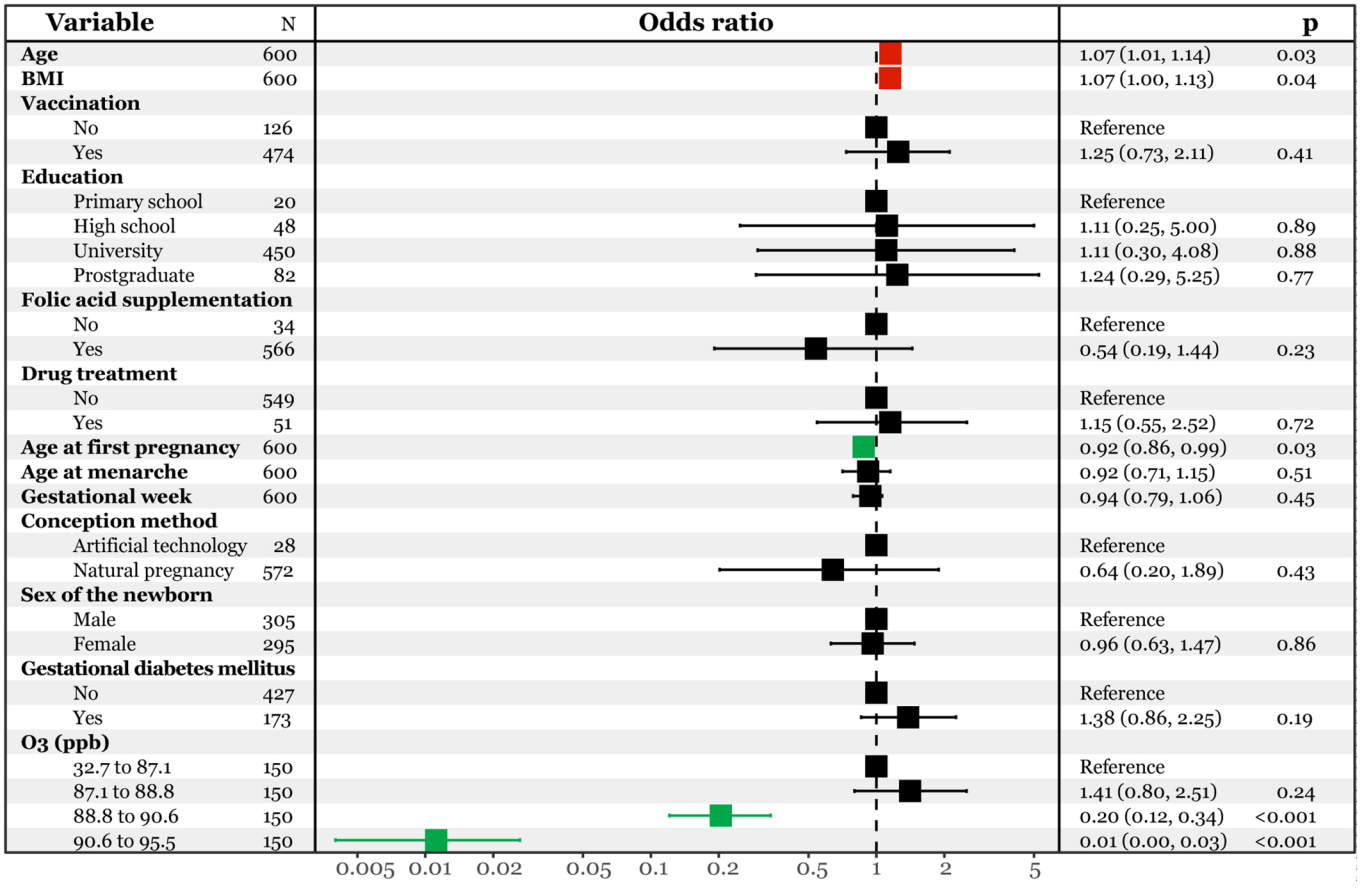
Multivariate logistic regression analysis predicting SARS-CoV-2 infection with interquartile ozone.

Moreover, we categorized the ozone data into four quartiles based on previous studies (Li et al., 2021; Qiu et al., 2021) and incorporated them into the multivariate logistic regression model (**Figure 3**). Compared to the first quartile (32.7 to 87.1 ppb), the third (88.8 to 90.6 ppb) and fourth (90.6 to 95.5 ppb) quartiles were associated with a significantly lower risk of infection [OR95%CI: 0.20(0.12, 0.34) and 0.01(0.00, 0.03)]. Additionally, an increase in age at first pregnancy was linked decreased risk of infection [OR95%CI: 0.92(0.86, 0.99)], while an increase in age at pregnancy and pre-pregnancy BMI corresponded to a higher risk [OR95%CI: 1.07(1.01, 1.14) and 1.07(1.00, 1.13)]. The robustness of these two models was confirmed through receiver operating characteristic (ROC) analysis, yielding area under the curve (AUC) values of 0.842(0.809, 0.876) (**Supplementary Figure S2**) and 0.860(0.830, 0.890) (**Supplementary Figure S3**).

## 4 Discussion

To study the effect of longitudinal gestational ozone exposure on respirovirus infection in late pregnancy, we conducted this retrospective cohort study, which involved 600 pregnant women who gave birth to singletons in Jinan, China, between January 2022 and March 2023. The atmospheric ozone levels in this study area were meticulously measured, along with individual ozone exposure levels during each trimester. Comprehensive data collection was undertaken, encompassing demographic, clinical, and laboratory data of the expectant mothers and their newborns. The findings revealed a concerning deterioration in Jinan’s atmospheric ozone, exhibiting fluctuations in sync with local temperature and wind directions. Interestingly, an inverse association was discovered between gestational ozone exposure and late pregnancy SARS-CoV-2 infection after adjusting for potential confounders, and this intriguing finding hints at a possible protective role of ozone against SARS-CoV-2 infection in pregnant women.

While the precise mechanism of ozone’s antiviral activity remains to be fully elucidated, it is postulated that ozone may interact with viruses via a direct molecular ozone reaction pathway and/or indirectly through the generation of reactive oxygen species (ROS) such as hydroxyl radicals (·OH), superoxide anions (O2·-), and hydrogen peroxide (H2O2) as byproducts of ozone decomposition (Tizaoui, 2020). In line with previous studies, the application of ozone to inactive SARS-CoV-2 had been well documented (Bayarri et al., 2021; Yano et al., 2020). For example, Angeles et al. reported that ozone can oxidize the viral envelope and RNA, rendering the virus unable to infect host cells (Blanco et al., 2021). Particularly, previous studies have demonstrated the inactivation of a human coronavirus (HCoV-229E) and other related viruses by ozone under various conditions (Lee et al., 2021). However, ozone is also a hazardous gas that can cause lung damage if inhaled at high levels, and therefore strict safety protocols must be followed when using ozone as a disinfectant.

As a potent oxidant, ozone has been demonstrated to inactivate SARS-CoV-2 through a multi-faceted mechanism. For instance, it can inflict damage on the viral envelope, a lipid membrane that safeguards the virus’s genetic material, which houses proteins vital for the virus to latch onto and infiltrate host cells, while ozone’s oxidizing properties can impair these proteins, thereby obstructing the virus’s ability to infect new cells (Tizaoui, 2020). Furthermore, ozone can permeate the viral envelope to reach the viral genome, composed of RNA. By inducing breaks and mutations in the RNA, ozone incapacitates the virus’s ability to replicate or express its genes (Epelle et al., 2023; Farooq and Tizaoui, 2023; Tizaoui, 2020). The cumulative impact of ozone on the viral envelope and genome culminates in the inactivation of SARS-CoV-2 and other enveloped viruses, suggesting that atmospheric ozone could potentially serve as a natural deterrent against SARS-CoV-2 infection in pregnant women.

Moreover, the application of ozone for COVID-19 therapy has shown promising results in various studies. A prospective case-control study demonstrated that ozone therapy was associated with lower mortality, shorter hospital stay, and improved oxygenation in patients with COVID-19 pneumonia (Hernández et al., 2021). Furthermore, ozone has been found to be effective in inactivating airborne viruses, including a surrogate for SARS-CoV-2, in a wind tunnel simulating swine building, achieving more than 3 log10 reduction of viral infectivity within 6 minutes at concentrations of 0.5 to 1.8 ppm (Vyskocil et al., 2020). Additionally, a study showed the feasibility of using ozone generated by a plasma device to disinfect face masks contaminated with SARS-CoV-2, where ozone at a concentration of 6 ppm could inactivate more than 99.9% of SARS-CoV-2 within 55 minutes (Lee et al., 2021). Consistently, we found protective effects of gestational ozone exposure on late pregnancy SARS-CoV-2 infection, which suggests that ambient ozone may directly participate in the clearance of SARS-CoV-2, or indirectly enhance the immunity of pregnant women and increase the resistance of relevant populations to SARS-CoV-2. Besides, Bernardino et al. proposes that ozone therapy could be used as an alternative or complementary therapy in the different phases of SARS-CoV-2 infection, depending on the dose and route of administration (Clavo et al., 2020). They emphasize the rectal insufflation technique as a simple, safe, and inexpensive method of systemic ozone delivery. However, it calls for urgent experimental studies to confirm or rule out the biological effects of ozone therapy and to evaluate its safety and efficacy for the management of SARS-CoV-2 infection.

Currently, ozone therapy has been explored for its potential to mitigate oxidative stress and exert antiviral effects, which could be especially beneficial during pregnancy. Studies have shown that ozone can reduce viral load, regulate oxidative stress, and balance the immune response, thus offering a potential therapeutic approach for managing infections like COVID-19 (Izadi et al., 2021; Ogut and Armagan, 2023; Radvar et al., 2021). While evidence is still emerging, ozone therapy’s effects on pregnant women require further investigation to ensure its safety and efficacy. In particular, the regulation of oxidative stress by ozone could be crucial in reducing the adverse outcomes associated with SARS-CoV-2 infections during pregnancy. Notably, ozone therapy has been used in adults for various, typically through methods like autohemotherapy, where controlled doses are administered (Harapan and Harapan, 2023). However, there is limited evidence regarding the safe use of ozone in neonates and pregnant women. The literature suggests caution in administering ozone therapy during pregnancy due to potential risks to both the mother and fetus. Controlled ozone administration may be a possibility for pregnant women, but further studies are needed to determine the appropriate dosages and safety protocols for this population.

Although ozone therapy has shown promise as an antiviral treatment, it is important to note the potential risks associated with ozone exposure, particularly in high concentrations. Ozone is a potent oxidant that can cause respiratory irritation, damage to lung tissue, and other health issues when inhaled in large amounts (Shekhar et al., 2021). For pregnant women, the risks of ozone exposure are magnified due to the potential harm to both maternal and fetal health (Chen et al., 2023; Zhang et al., 2024). Therefore, strict safety protocols and controlled ozone administration are essential to minimize these risks and ensure the safe use of ozone therapy in treating COVID-19 and other viral infections.

In the context of longitudinal ozone exposure over gestation and late pregnancy SARS-CoV-2 infection, both factors may affect the maternal immune system and fetal development in different ways. However, previous studies have also linked longitudinal ozone exposure over gestation to increased risks of preterm birth, low birth weight, intrauterine growth restriction, preeclampsia, and congenital anomalies. For example, late pregnancy SARS-CoV-2 infection is demonstrated to be associated with increased risks of severe maternal illness, preterm delivery, cesarean section, neonatal intensive care unit admission, and vertical transmission (Di Toro et al., 2021; Ferrazzi et al., 2020; Knight et al., 2020). Therefore, exploring the interaction between longitudinal ozone exposure over gestation and late pregnancy SARS-CoV-2 infection is crucial, which may contribute to the definition of an appropriate dose of gestational ozone exposure.

Additionally, our findings suggest that higher levels of ozone exposure during pregnancy may reduce the risk of SARS-CoV-2 infection in late pregnancy. However, the applicability of these results to regions with different ozone levels remains uncertain. In areas with elevated ozone concentrations, there may be a different immune response to viral infections compared to regions with lower ozone levels. Further research is required to explore the impact of geographic variations in ozone exposure on COVID-19 susceptibility, particularly in populations with differing environmental conditions.

There are several strengths and limitations of this study. The strengths include the robust sample size of 600 pregnant women, extensive data collection from electronic medical records, validated ozone exposure assessment based on hourly measurements, and adjustment for multiple confounders in the statistical analysis. However, there are some limitations that should be considered when interpreting the findings. First, the study was conducted at a single hospital in Jinan, China, which may limit the generalizability of the results. Second, ozone exposure was estimated based on ambient monitoring data rather than personal measurements, which may misclassify individual-level exposures. Third, the assessment of COVID-19 status was based on PCR testing at a single timepoint, which could underestimate the true prevalence of SARS-CoV-2 infection. Fourth, the retrospective design precludes establishing a temporal relationship between ozone exposure and COVID-19 onset. Finally, residual confounding by unmeasured factors cannot be ruled out. Overall, this study provides initial evidence on the association between gestational ozone exposure and SARS-CoV-2 infection, but additional longitudinal studies with more rigorous exposure assessment are warranted to confirm the findings.

## 5 Conclusions

To sum up, this retrospective cohort study found an inverse association between longitudinal ozone exposure over gestation and the risk of SARS-CoV-2 infection in late pregnancy among 600 pregnant women in Jinan, China. After adjusting for demographic and clinical factors, higher ozone exposure throughout pregnancy was associated with a significantly lower likelihood of testing positive for COVID-19 in the third trimester. However, this study is limited by its retrospective design and reliance on ambient ozone measurements, and hence further longitudinal investigations with more rigorous ozone exposure assessment at the individual level are warranted to validate the observed association between gestational ozone exposure and SARS-CoV-2 infection risk.

## Data Availability

The original contributions presented in the study are included in the article/**Supplementary Material**. Further inquiries can be directed to the corresponding author.
