# Supplementary material for: Longitudinal ozone exposure and SARS-CoV-2 infection in late pregnancy: a retrospective cohort study
Source: Front Cell Infect Microbiol. 2024 Dec 20;14:1476603. doi: 10.3389/fcimb.2024.1476603 (PMC11695408; doi:10.3389/fcimb.2024.1476603)
Supplement: Supplementary file 1 [file Table1.docx]

**Supplementary materials**


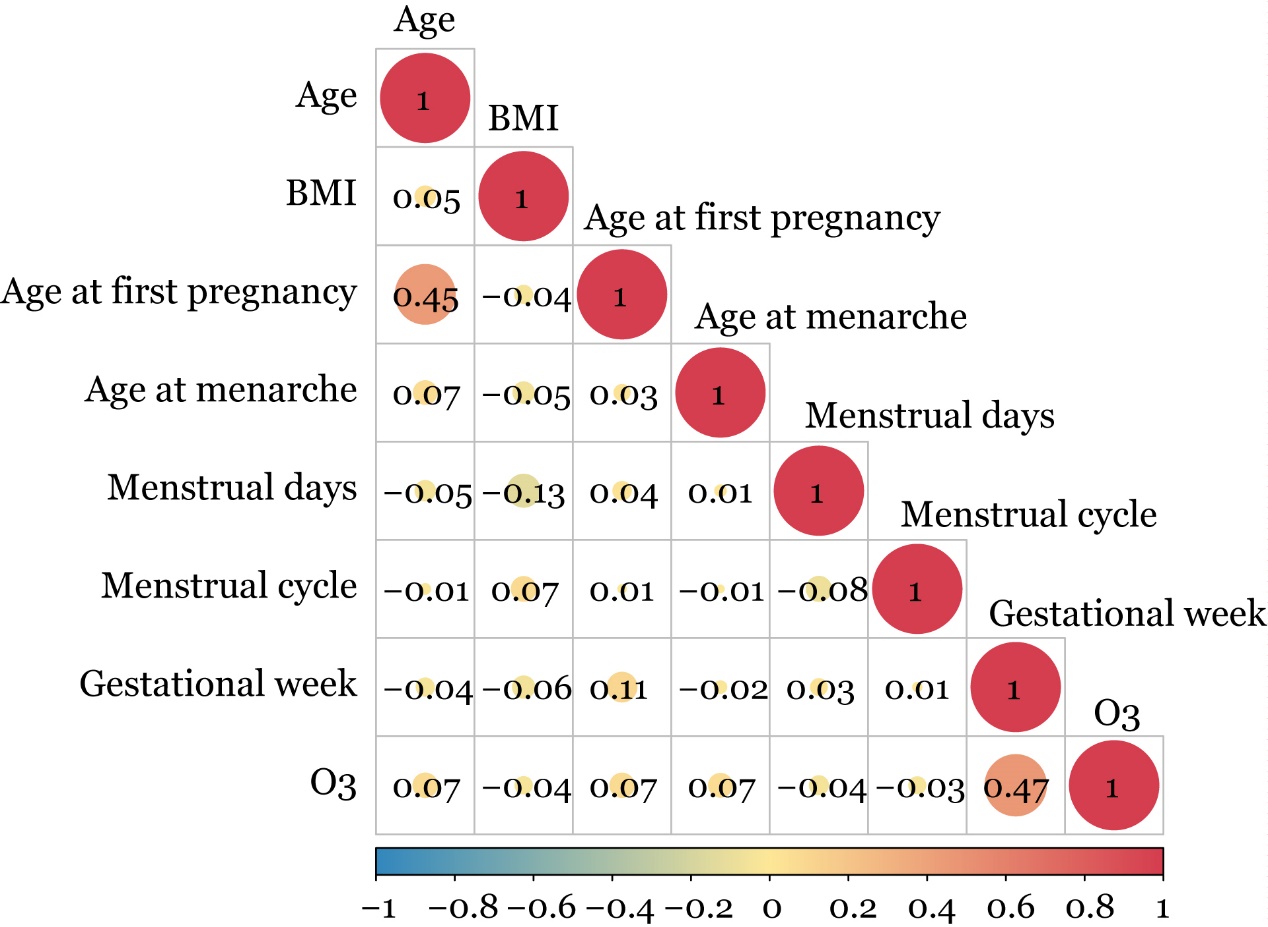


**Figure S1**. Correlation analysis between ozone and demographical variables.


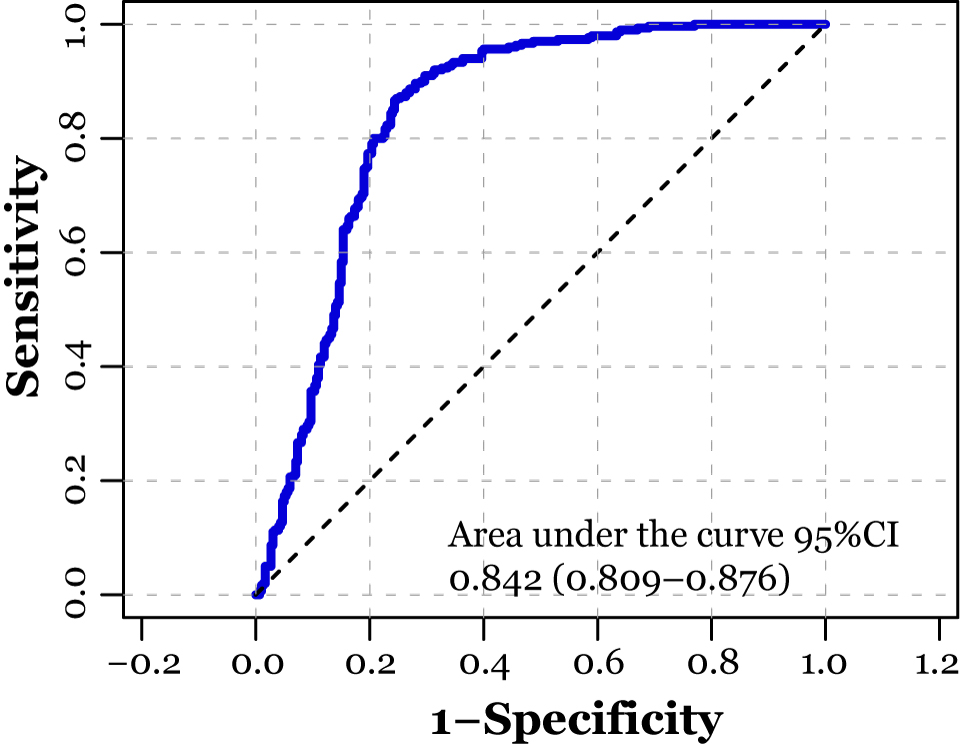


**Figure S2.** Receive operation cure analysis of multivariate logistic model with continuous ozone
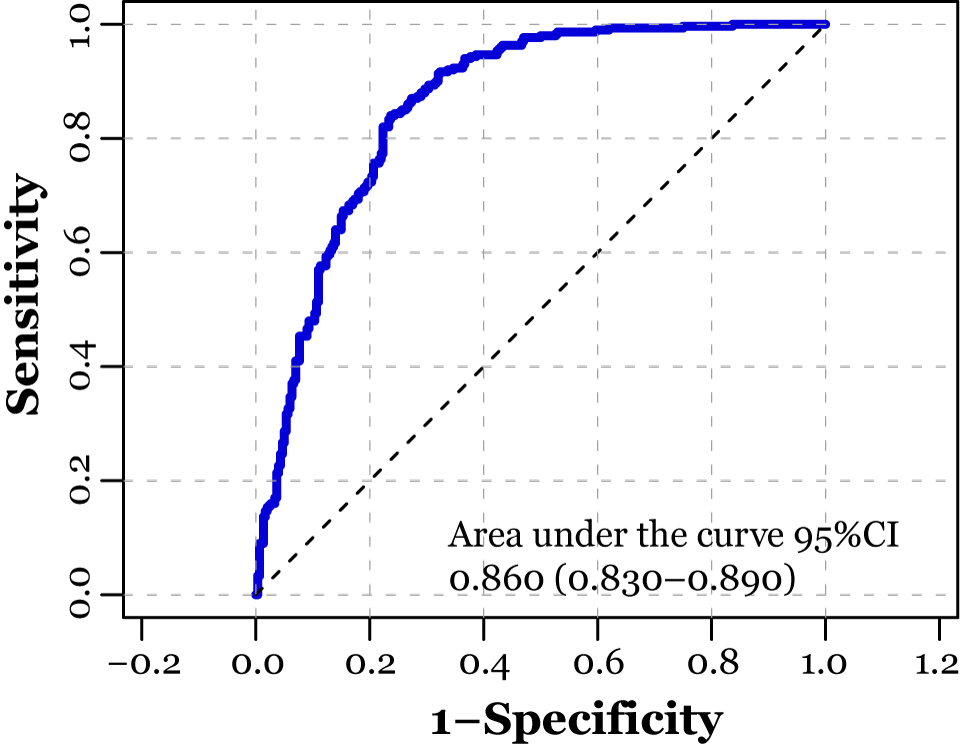


**Figure S3.** Receive operation cure analysis of multivariate logistic model with interquartile ozone
